# Leveraging principal component analysis to uncover urban pedestrian dynamics

**DOI:** 10.1007/s10109-025-00469-0

**Published:** 2025-06-10

**Authors:** Jack Liddle, Wenhua Jiang, Nick Malleson

**Affiliations:** 1 School of Geography, University of Leeds, Leeds, UK; 2 Alan Turing Institute, London, UK

**Keywords:** Principal component analysis, Footfall, Urban dynamics, Geographic information science, C38, R41, R23, O18, P25, R00, C88

## Abstract

As the world rapidly urbanises and cities become larger and more complex, understanding pedestrian dynamics is paramount. New data sources, particularly those that measure pedestrian counts (i.e. ‘footfall’), offer potential as a means of better understanding the fundamental spatio-temporal structures that characterise aggregate pedestrian behaviour. However, footfall data are often complex and influenced by a wide range of social, spatial and temporal factors, which complicates interpretation. This paper applies principal component analysis (PCA) to hourly pedestrian count data from Melbourne, Australia, to extract the key temporal signatures that underpin observed urban footfall patterns. PCA can reduce the dimensionality of noisy pedestrian flow data, revealing dominant activity patterns such as weekday commuting cycles and weekend leisure activities. By subsequently analysing pedestrian volumes through the lens of these components, we start to expose the underlying types of pedestrian activities that characterise different neighbourhoods. In addition, we can distinguish multiple overlapping activity patterns within a single location, identifying changes in urban functionality and detecting shifts in mobility trends. The impacts of external shocks, such as the COVID-19 pandemic, are particularly stark. These findings shed light on the intricacies of urban mobility and suggest that there is value in the use of PCA as a means to better understand urban dynamics.

## Introduction

As the world becomes increasingly more urbanised—by 2050, 68% of the world’s population is projected to live in urban areas (United Nations 2018)—understanding pedestrian dynamics is crucial. The importance of pedestrian activities in creating safer, more vibrant cities has been discussed for some time (Jacobs 1961). However, only recently has the “golden age of data” (Arribas-Bel and Tranos 2018) enabled large-scale quantitative analyses of pedestrian dynamics. This has fostered a greater awareness of the need for data-driven empirical evidence (Philp et al. 2022) to support urban development.

**Fig. 1:**
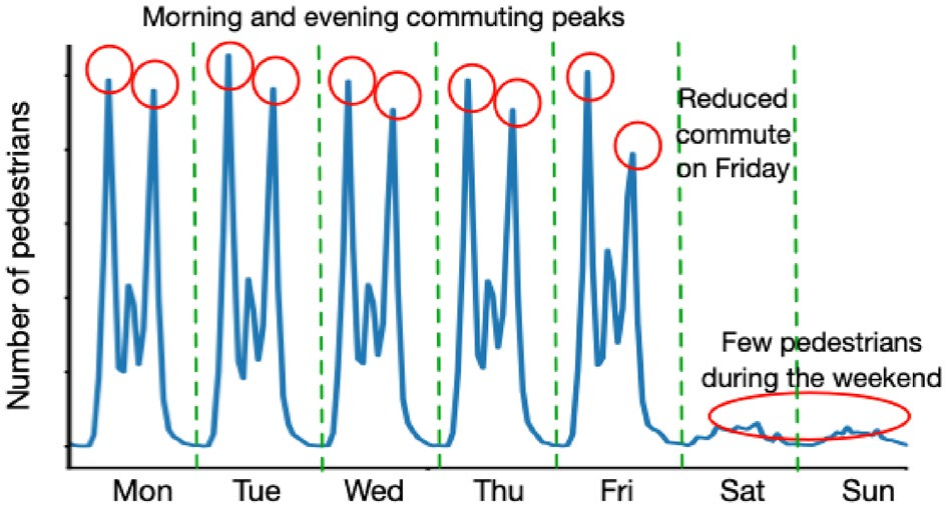
Hypothetical footfall counts for an urban location over a week. The location exhibits peaks in the morning and evening during weekdays that are likely to correspond to people commuting. During the weekend, there is substantially lower footfall, suggesting that this may be a place that does not attract visitors for activities other than work

Despite this, there has been relatively little attention paid to the *temporal signatures* that emerge from the activities of pedestrians in places. These signatures that provide estimates for the changing number of pedestrians who were present in a particular place over a particular time period can reveal insight into the evolving usage patterns of the built environment over short (hourly) or longer (weekly/yearly) time scales. For example, Fig. 1 illustrates hypothetical footfall counts for an urban location over the course of a week. Qualitatively, the counts appear to suggest that the vast majority of people who visit this area will do so for the purposes of traditional ‘9–5’ employment. However, without a more formal quantitative assessment of footfall dynamics we cannot answer questions such as: are there additional hidden ‘signals’ present that are suppressed by the dominant commuting pattern?; is this kind of pattern representative of the dynamics present in other places, or is it unique?; does this pattern change over the course of a year, or has it changed noticeably over the last decade? (which would indicate an evolution in the activities undertaken in the area); etc. Ultimately, without isolating the individual temporal signatures that, together, make up the observed footfall counts, we might miss some of the key pedestrian dynamics that underpin wider urban processes.

Gaining such insight from pedestrian counting datasets can be extremely difficult. Footfall is influenced by both macro-scale effects (economic trends, weather conditions, a pandemic, etc.) and micro-scale effects (the presence of particular shops, the perceived ‘character’ of a place, the configuration of transport infrastructure, etc.) (Philp et al. 2022) which results in the presence of a very large number of diverse factors that influence the flows of pedestrians in different ways, both spatially and temporally. Recognising the regular signals that emerge from so much noise can be challenging. However, dimensionality reduction can help to simplify these temporal data, enhancing the clarity of long-term trends and cyclical behaviours inherent in pedestrian movements.

To begin to answer questions such as those above, this paper presents a formal, quantitative approach to the analysis of temporal footfall patterns. The aim is to extract the core temporal ‘signatures’ that underpin otherwise noisy and diverse pedestrian count data and can explain most of the variability across the city. We leverage hourly pedestrian count data—an important innovation in itself as hourly variations are often overlooked in footfall studies—and use principal component analysis (PCA) to reduce otherwise complex footfall counts into a small number of principal components that encapsulate the most significant variations in footfall. Interestingly, the components that explain most of the variance are easily recognisable as being related to common activities, such as commuting peaks in the mornings and evenings, or activities during the day on weekends. By then further analysing these components themselves we can start to identify different area types—i.e. those that are characterised largely by commuting activities v.s. those that are more likely to be associated with daytime leisure activities—and identify how the usage patterns in different parts of the city vary over time.

It is worth noting that exploratory data analysis (EDA) techniques are also commonly used to explore footfall patterns, yet they are limited in their ability to systematically decompose complex, overlapping temporal activity patterns. PCA offers distinct advantages by quantitatively isolating distinct *components*. This allows us to: (i) extract dominant temporal signatures, such as weekday commuting cycles or weekend leisure activities, in a way that avoids subjective interpretation; (ii) quantify variations in the dominance of different signatures across locations and time periods in a systematic way; and (iii) identify relationships between recurring patterns (such as changes in commuting behaviour that might take place alongside other changes in a location) that can be obscured in simpler visual analyses. These benefits make PCA particularly suited to uncovering latent patterns in pedestrian dynamics that would be difficult to extract using EDA alone.

The main contributions of the paper are threefold:

1. We demonstrate that, using PCA, it is possible to simplify and distil complex, noisy pedestrian count data into a small number of interpretable components that represent the core temporal footfall signatures. This reveals the dominant temporal patterns in urban pedestrian activities.
2. By clustering the principal components that represent the temporal footfall patterns in a particular area, it is possible to isolate quantitatively different footfall patterns that exist at that location. This provides strong evidence that some areas attract people for multiple different purposes as distinct from others that exhibit similar usage patterns throughout the week.
3. By examining the changes in the scores of the principal components over time, we can highlight the presence of latent factors that drive longer-term changes in pedestrian mobility patterns. Specifically the paper identifies shifts in urban functionality, particularly as a result of the COVID-19 pandemic, that suggest that the activities that people engage in when they visit some areas have materially changed.

The paper is structured as follows: Sect. 2 reviews the relevant literature; Sect. 3 outlines the methodology and the main method used (PCA); Sect. 4 outlines the data sources used and conducts an exploratory data analysis; Sect. 5 discusses the PCA implementation; Sect. 6 presents and discusses the results; and Sect. 7 draws conclusions.

## Previous work

Quantifying footfall—i.e. the number of pedestrians moving through a place at a particular time—is crucial for applications as diverse as urban planning (Cooper et al. 2021), economic strategy (Mumford et al. 2021; Philp et al. 2022), environmental health (Park and Kwan 2017), and public safety (Malleson and Andresen 2016; Boivin and Felson 2017; Hanaoka 2018; Tucker et al. 2021). Recent years have seen the emergence of a literature on “ambient” (Whipp et al. 2021), “day time” (Boeing 2018) and “temporary” (Charles-Edwards and Bell 2013; Panczak et al. 2020) populations. These refer to different measures of the dynamic populations present in an area, i.e. commuters, shoppers, students, tourists, event attendees, etc. For a recent review of the ‘ambient’ population literature, the interested reader can refer to Panczak et al. (2020) or Richardson (2020). However, with a few exceptions (Charles-Edwards and Bell 2013; Ma et al. 2017; Liu et al. 2018; Richardson 2020), much less attention has been paid to the hour-by-hour changes that occur in specific locations that emerge from the activities of pedestrians in places. One reason for this is that, historically, residential-based data have been much more forthcoming than footfall data. Fortunately, in recent decades, a range of data sources have become available that either provide proxy estimates of footfall, or count people directly. These include, for example, telecommunications data (Traunmueller et al. 2014; Bogomolov et al. 2014; Song et al. 2023), smartphone apps that capture mobility traces (Richardson 2020), street view images (Chen et al. 2020, 2022), social media data (Malleson and Andresen 2015a, b; Botta et al. 2015; Liu et al. 2022), publicly available traffic videos (Dobler et al. 2021) and, importantly, devices that count pedestrians as they pass by a sensor (Kontokosta and Johnson 2017; Crols and Malleson 2019; Soundararaj et al. 2020; Philp et al. 2022).

One of the most common application areas for the study of high-resolution footfall data is in the area of retailing. To measures their success, shops need to estimate the number of potential shoppers who pass their premises. This is even more important in an age where physical businesses are competing with online retailers and, more broadly, because footfall is the “lifeblood” (Philp et al. 2022) of a high street. For example, Trasberg et al. (2021) show that the inclusion of footfall data into models that predict store sales improves their predictive capability, highlighting the importance of footfall for store turnover. In a similar vein, Philp et al. (2022) use pedestrian foot traffic in retail environments to classify micro-locations into distinct clusters that are related to their retail characteristics.

Beyond retailing, the analysis of footfall data is also relevant for understanding urban dynamics more broadly. For example, Dobler et al. (2021) parse publicly available pedestrian traffic videos to estimate footfall counts in New York City and use these to explore the dynamics of pedestrian activity. As expected, they find a typical ‘3-peak structure’ (morning commute, lunch time, evening commute) in weekday pedestrian behaviour and a steadier change over weekends. These results are strikingly similar to those presented in this paper, although here we go further by trying to isolate the different ‘signatures’ that lead to the emergence of the overall observed patterns.

Methodologically speaking, very few papers have tried to derive insight into the underlying temporal signatures that make up aggregate footfall patterns. In this paper, we approach the problem using principal component analysis (PCA) as a way to reduce the complexity of noisy footfall data into a few core statistical components. Although widely used in general, there are relatively few applications of PCA to studies of pedestrian dynamics. Chraibi et al. (2016) use functional PCA (a variation of PCA that can be applied to trajectory data) to validate their agent-based pedestrian models, comparing simulated outputs with real data. Their work is relevant here because they argue that PCA is a valuable tool for offering insight into pedestrian dynamics (although focusing on count data rather than individual trajectories), which aligns with our aims. More broadly, the functional approach (e.g. Ramsay and Silverman 2006) has been used to study temperature and precipitation (Ramsay and Dalzell 1991) as well as neighbourhood change (Jung and Song 2022). Such an approach may be appropriate here because it would treat the footfall data as continuous functional data, making the approach better suited to handling temporal continuity. However, for this preliminary study we use ‘traditional’ PCA as it is more likely to produce clear, explainable components, which is more important for understanding footfall patterns than generating optimal components.

A related approach that could have been employed, rather than PCA, is independent component analysis (ICA). ICA attempts to identify statistically independent components, focusing on separating mixed signals into distinct sources (Hyvärinen and Oja 2000). This approach is well-suited for scenarios where the goal is to separate mixed signals or to identify independent sources of variation, such as separating audio signals. However, temporal patterns in time-series pedestrian data, such as daily and weekly cycles, are typically correlated as they represent recurring trends over time. For example, footfall data may show strong correlations between certain hours of the day or between weekdays and weekends. These patterns are periodic and follow a predictable structure, rather than being independent signals. Therefore, ICA’s focus on independence, rather than correlation, makes it less suitable for capturing such temporal patterns. In addition, solving ICA optimisation, which is inherently non-convex, from different initial approximations can lead to varying solutions, making it harder to identify the optimal number of components that underpin regular pedestrian behaviour and can be interpreted qualitatively to better understand urban dynamics (Tibaduiza et al. 2012; Kairov et al. 2017). Similar observations have been noted in prior studies using PCA for temporal analysis (e.g. Jolliffe 1986; Abdi and Williams 2010.

We also considered the use of nonnegative matrix factorisation (NMF). NMF is one of the most widely used dimensionality reduction techniques and is particularly effective for handling nonnegative data (such as pedestrian counts). Unlike PCA, which maximises variance, NMF focuses on minimising reconstruction error (Gan et al. 2021). However, NMF raises similar problems to those of ICA in that the approach to determining the optimal number of components is not clear (Cai et al. 2022; Maisog et al. 2021). On the other hand, with PCA the first few components explain the most variance which allows for the extraction of dominant temporal patterns that aligns with our objective of uncovering key urban footfall dynamics.

The most directly similar study is that of Kim (2020). The authors use cell phone activity count data (aggregated to a 50 m^2^ spatial grid and hourly temporal bins) as a proxy for pedestrian dynamics and apply functional PCA to those data to explore characteristics of ‘urban vitality’. While there are similarities in the components identified by Kim, such as the aforementioned ‘3 peaks’, there are also notable differences. For example there is the absence of any strong ‘lunch time’ behaviour (that we uncover later in Fig. 8), although this may be because the authors only present the shapes of the first two components. Finally, Elhaik (2022) caution that PCA-derived results may not be “reliable, robust, or replicable.” However, their findings are specific to genetic studies, and it remains unclear whether these issues apply more broadly. Their assessment is not echoed in other PCA-related studies.

## Methodology

### An overview of PCA

Principal component analysis (PCA), originally developed by Hotelling (1933), is a statistical technique used for dimensionality reduction. It aims to analyse a data table, where each row contains a number of inter-correlated observations (termed ‘variables’), and transform the original table into a new set of variables that summarise the most significant features. These features, referred to as the ‘principal components’, are constructed in such a way that they are uncorrelated. This is achieved through orthogonality: the components are at right angles to each other in a multi-dimensional space. Once the components have been established, the original observations can be approximated through a linear combination of the principal components and a set of unique *loadings* (i.e. coefficients) that are applied to each component. Section 3.2 explains this process in more detail in the context of PCA for footfall data.

The quality of the PCA—i.e. the extent to which the raw data can be reconstructed purely through a linear combination of the components—can be quantified using the *proportion of the explained variance*. This represents the proportion of the total variance in the data set that is captured by each principal component (Elhaik 2022). The first component will have the largest possible variance and hence will ‘explain’ the largest part of the variation observed in the data table, with subsequent components explaining gradually less of the variance. The proportion of the explained variance is calculated by dividing the variance captured by a specific principal component by the total variance of the original data set. This metric is crucial for understanding how much information from the original data set is retained after the dimensionality reduction process and can be used, as we do here, to determine how many components need to be retained in order to reconstruct the original data to an acceptable degree of accuracy.

For a fuller outline of PCA and worked example, the interested reader can refer to Abdi and Williams (2010) or Jolliffe (2011). The remainder of this section outlines the process of applying PCA to hourly pedestrian count data from Melbourne, Australia.

### PCA for the footfall data

The data used here encompass measurements from 94 sensors distributed across Melbourne, recording the number of people who walk past each sensor in hourly intervals. Full details of the data collection and pre-processing steps are provided in Sect. 4.

We consider two periodicities for our analysis: days and weeks. These are chosen because they capture the most important features of typical urban dynamics, but future work might also consider aggregation by alternative periodicities such as months or seasons (winter, summer, etc.). To explain how PCA works in our context, consider just the daily periodicity. In this case the footfall pattern for a particular sensor on a particular day is encoded as a 24-item vector where each item represents the counts for each hour in the day. We refer to a sensor-day vector as an ‘observation’. Each observation can therefore be thought of as a single point in a 24 dimensional space, and the entire data table becomes a 24-dimension point cloud, where the number of points (observations) is equal to the number of sensors multiplied by the total number of days in the study time period. The principal components can then be thought of as *directions* in that space, each necessarily represented by a 24-item vector. The components are organised such that the first explains most of the variation in the points, with latter components explaining iteratively less variation. The original observations can then be approximated through a linear combination of the principal components and their loadings (i.e. coefficients). Loadings can be positive or negative, with negative loadings effectively reversing the influence of a component on an observation.

As a raw observation is a 24-item vector, if we conducted a PCA with 24 components then each observation in the original data could be recreated perfectly. This is not useful though, so the aim of PCA is to reproduce the original data reasonably well with the use of fewer than 24 components, i.e. *dimensionality reduction*. The PCA process is identical for the weekly aggregation, except that the space is larger with $24 * 7 = 168$ dimensions.

**Fig. 2:**
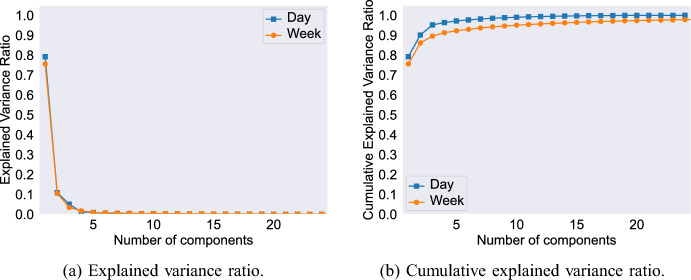
Explained variance ratio and cumulative explained variance of PCA for daily and weekly aggregation

To identify the most suitable number of components to consider, Fig. 2a illustrates the explained variance ratio for each component under daily and weekly aggregations. For both aggregations we see that a large amount of the variation in the data can be explained by the first component and that there are diminishing returns from the fourth component onwards. Observing the cumulative explained variance ratio (Fig. 2b), it is apparent that, with only three components, 95% of the variation for the daily aggregation and 90% of the variation for weekly aggregation can be captured. Therefore, in the later descriptive results analyses (Sect. 6) we concentrate on the first three components as these represent the clearest temporal signatures and explain most of the observed variation.

## Data

### Melbourne pedestrian counters

**Fig. 3:**
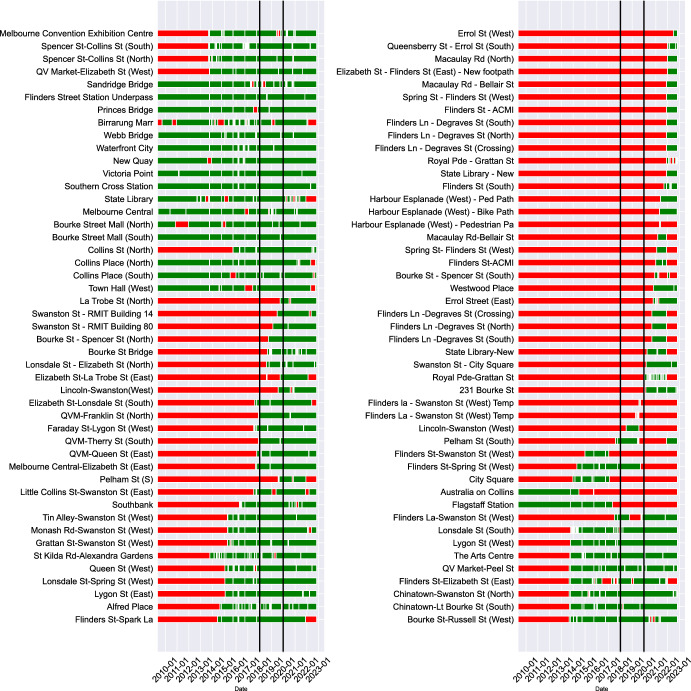
Graph illustrating the days that each sensor reports at least one count (green) or no counts (red) (colour figure online)

Although a number of cities across the globe publish pedestrian count data, the Melbourne data set^1^ is by far the most comprehensive in terms of both total sensor numbers and period of time available. The data set contains hourly pedestrian counts at numerous locations across the city, covering more than a decade with the earliest records being made in May 2009. There are 94 sensors that have been active at some time from 2009 to the present, although not all sensors were active initially and have not necessarily remained active up to the time of writing. Figure 3 illustrates the time periods in which each sensor has returned at least one pedestrian count value. In this study we analyse two full years of data from the beginning of 2018 to the end of 2019. This period was chosen because it contains the largest volume of count data but is also not affected by the COVID pandemic that began in 2020 and significantly disrupted ‘normal’ urban activities.

**Fig. 4:**
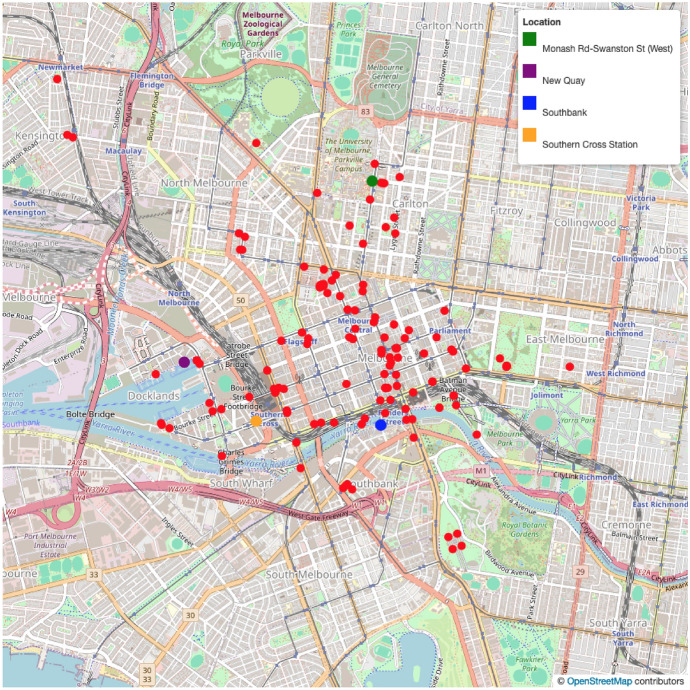
Sensor locations in the Melbourne data set and the locations of four case study sensors

The sensors are located across the city, sampling a wide range of different sites including retail and business/commercial zones as well as transportation hubs and leisure venues, as per Fig. 4. The sensors’ footfall detection mechanisms are likely to be based on a Doppler radar system^2^ although published details about the detection mechanisms and the rationale behind the spatial distribution of the sensors are opaque. The Doppler method counts the physical presence of a body in the space, avoiding a possible bias against the pedestrians who may be missed by systems that rely on detecting the presence of WiFi or Bluetooth signals from a pedestrian’s mobile phone (e.g. Kontokosta and Johnson 2017; Crols and Malleson 2019; Soundararaj et al. 2020 Trasberg et al. 2021.

### Data preparation

To prepare the data for PCA, we reshape the raw hourly counts into two alternative structures for daily and weekly analysis. In the reformatted datasets, each row corresponds to a single sensor-day or sensor-week observation, represented as a 24-column or 168-column vector of hourly counts, respectively. Table 1 illustrates the structure of these datasets. If a sensor has incomplete records for any given day or week (i.e. if even one hourly count is missing), then that day or week for that sensor is discarded.

**Table 1:**
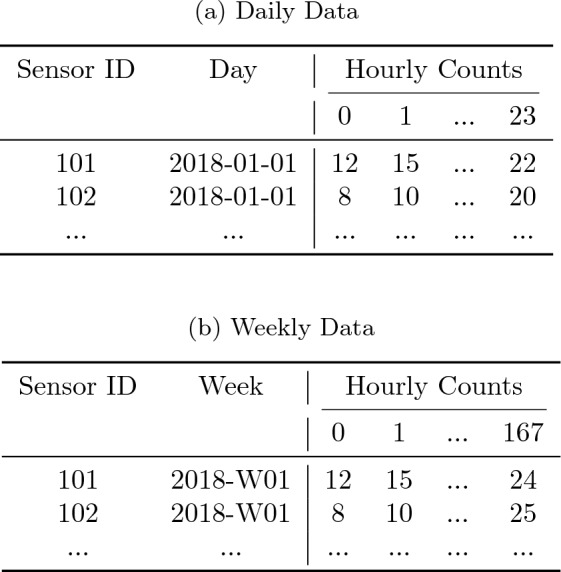
Illustration of reformatted data for PCA

**Fig. 5:**
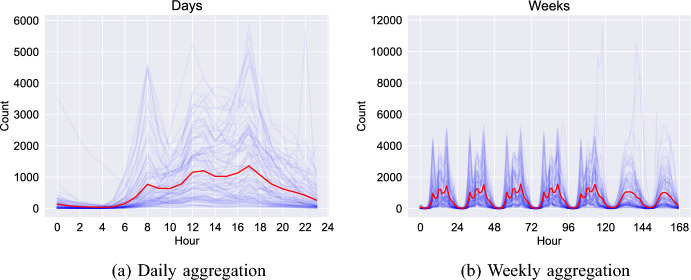
Aggregating on days and weeks. The mean is plotted in red as well as a random sample of 100 sensor-day/week pairs

The daily and weekly counts for 100 randomly chosen observations as well as the mean counts across all observations are shown in Fig. 5. In Fig. 5a (daily aggregation) we can see a typical urban daily schedule (typical of many Global North cities at least), characterised by a quiet period during the early hours and three activity peaks corresponding to morning, midday and late afternoon. Similarly, Fig. 5b (weekly aggregation) exhibits the same daily pattern repeated with a slightly different behaviour on the weekend; the three activity peaks are no longer visible. Overall, Fig. 5 suggests that there are some diverse activity patterns that can be distinguished, e.g. morning/evening commute, lunch time activities, etc. We would like to use PCA to try to extract and interrogate these quantitatively.

### Four case study locations

The aim of this paper is to isolate and analyse the key temporal signatures that comprise aggregate daily pedestrian activity across the city. To demonstrate the utility of our approach, we identify four sensors that are situated in locations that have particularly distinctive features of the built environment and, as a result, noticeably different aggregate footfall patterns. These locations were depicted in Fig. 4 and will be used in throughout the paper to show that the key elements that we extract using PCA can uniquely describe these neighbourhoods. The sensors we consider are:

*Monash Road–Swanston Street*: –Located at the in the centre of Melbourne University.
*New Quay*: –A riverside destination with leisure facilities.
*Southbank*: –Located at Southbank Promenade; a riverside destination featuring dining, arts, and leisure.
*Southern Cross*: –Southern Cross Railway Station, a major transportation hub serving intercity, local, and underground train services as well as bus services.

**Fig. 6:**
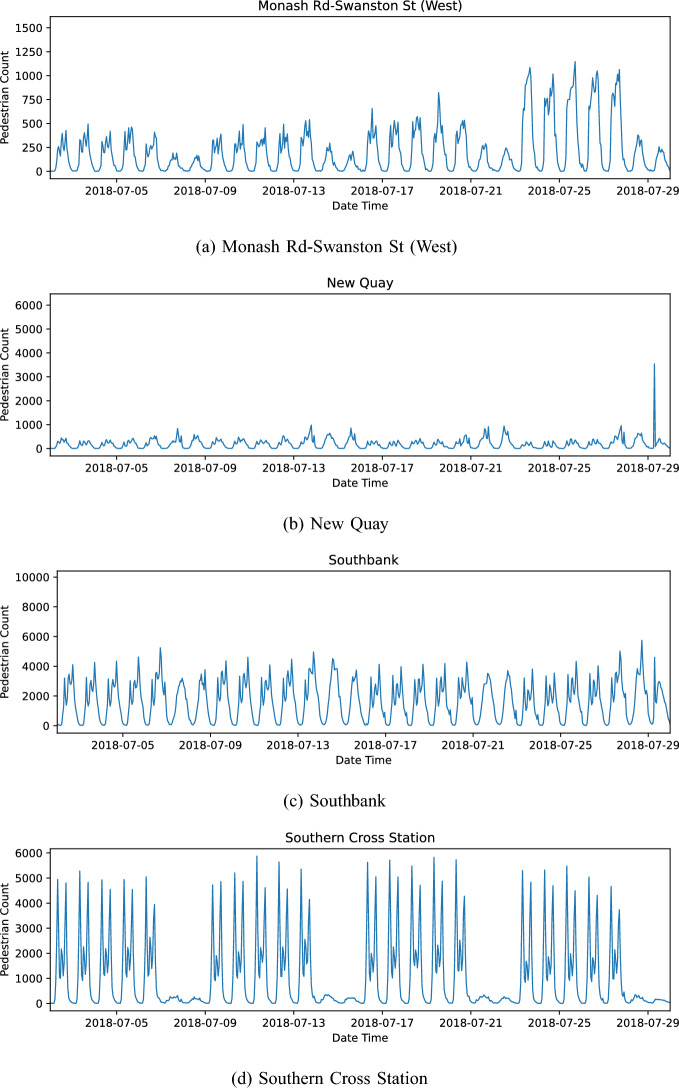
Hourly pedestrian counts for selected locations, over a single month. Note that the scale of the y-axis changes between the plots

To contextualise the case study locations, first consider the hourly count data over 4 weeks, shown in Fig. 6. All locations have a strong weekly periodicity. The daily periodicity is disrupted by the weekday/weekend cycle. The difference between the weekday and weekend footfall patterns varies between different locations, for example Southern Cross Station (Fig. 6d) shows a large drop-off in footfall during the weekends compared to Southbank (Fig. 6c). Within each weekday cycle we see three peaks corresponding to morning, noon, and afternoon. Within each daily cycle, the peaks vary in strength with Southern Cross Station (Fig. 6d) experiencing two strong peaks of equal strength during the morning and afternoon and with the relative height of the noon-peak being comparatively small. Contrast this with Monash Road (Fig. 6a) where the peaks are all of comparable height.

**Fig. 7:**
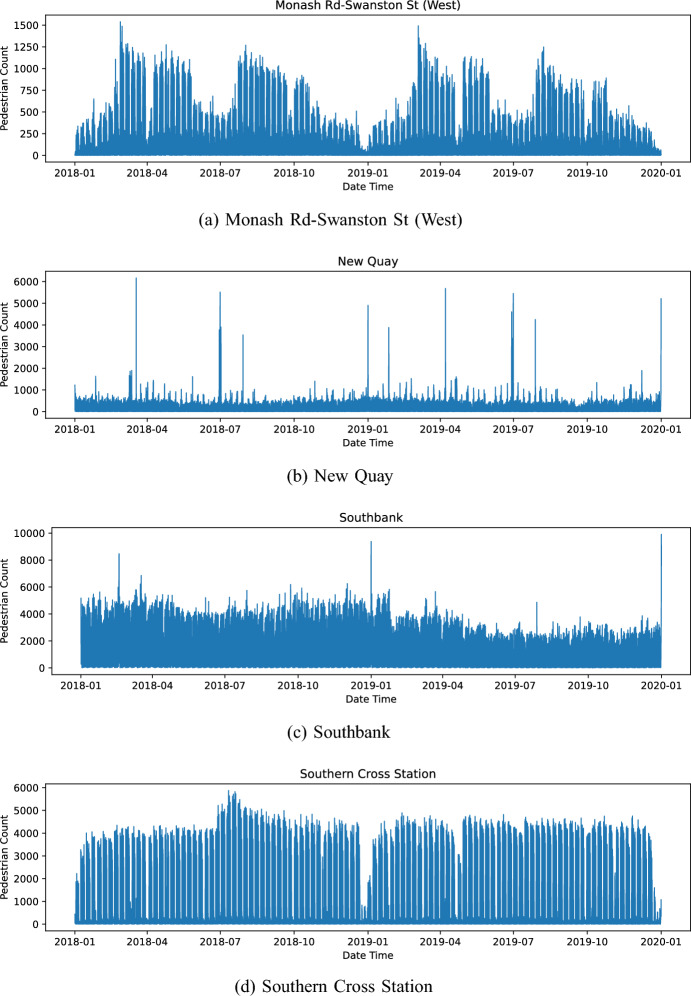
Hourly pedestrian counts for selected locations, over 2 years. Note that the scale of the y-axis changes between the plots

Looking at longer time scales, Fig. 7 reveals further complexities. Monash road, located in the centre of the University area, illustrates a complex interplay of drifts and multiple seasonalities (Fig. 7a). We see a strong weekly cycle with a greatly diminished pedestrian presence at the weekend and within each year we see two cycles corresponding to two university semesters. Additionally, within each semester we see a drop-off in attendance that corresponds to a reading week and a steady decline in footfall over each semester. Southbank (Fig. 7c) is located at Southbank Promenade, a riverside destination featuring dining, arts, and leisure activities, and shows a steady decline in pedestrian footfall over time with the year 2019 showing noticeably less footfall than 2018. New Quay (Fig. 7b) is another riverside destination with leisure facilities and demonstrates *anomalies* where there are large spikes in the pedestrian counts.

To summarise, Figs. 6 and 7 evidence the diversity of the footfall patterns and the likely related activities that occur throughout a city. Although we do not survey people to ascertain exactly what activities they are involved in, it is likely that these patterns distinguish activities like education, commuting, leisure, etc., as well as the overall ‘busyness’ of an area. It is extremely unlikely that any particular location will only exhibit one of these common activities; most places will contain a mix of activities that will not only vary throughout the week but may also change over longer time periods as the city develops and neighbourhood characteristics evolve. In the following section, we discuss our PCA implementation that we later use to extract these distinctive temporal features from aggregate pedestrian count data in order to better understand the nature of different parts of the city and how they might be evolving.

## Implementation and preliminary analysis

### Implementation of PCA

The analysis is conducted using the Python scikit-learn library and the source code is available in full on GitHub. The code automatically downloads the necessary data from the Melbourne Open Data portal as required. See the Data Availability Statement for full details. The PCA process itself is implemented in the sklearn.decomposition.PCA class.

### Labelling the principal components

**Fig. 8:**
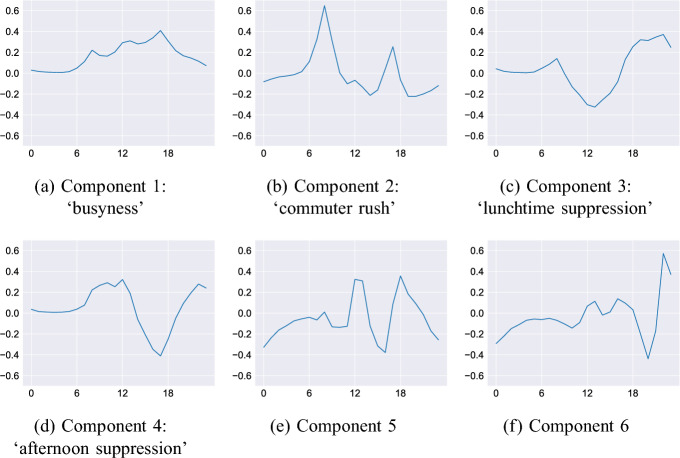
Principal components for daily behaviour

We conduct PCA on the daily and weekly aggregated data. Interestingly, the most important components (those that explain most of the variance in the original data) appear to be representative of different aspects of urban dynamics. Although this is not entirely unexpected, we were surprised that some of them exhibited such interpretable patterns. That said, the patterns quickly become harder to interpret so we only label the first four components. Starting with the daily aggregation, Fig. 8 plots the shapes of the first six of the principal components. Recall that for the daily aggregation each component is a vector of length 24, with each item representing a pedestrian count at a particular time.

- *Component 1, ‘busyness’ * (Fig. 8a). The low overnight counts and distinguishable peaks in the morning, midday and afternoon are almost identical to the mean daily activity exhibited in Fig. 5a. Therefore, this component does not represent any specific activity, but rather quantifies the average *busyness* of a location. An observation, *i*, with loading, $w_i > 0$ for this component will be busier than the average, and vice versa for those with $w_i < 0$.
- *Component 2, ‘commuter rush’ * (Fig. 8b). This component is distinguished by sizable peaks in the early morning and late afternoon that correspond closely to typical ‘rush hour’ commuting times. Observations with loadings $w_i > 0$ for this component will probably arise from sensors that are located in areas that are attended by large numbers of commuters.
- *Component 3, ‘lunchtime suppression’ * (Fig. 8c). This component represents a substantial decrease in footfall at around 12:00. Although it will also cause a slight increase in evening activity, peaking at approximately 20:00, the largest impact will be to reduce activity around lunch time.
- *Component 4, ‘afternoon suppression’ * (Fig. 8d). Similar to component 3, this component corresponds to a suppression of activity in the late afternoon and a slight increase at lunchtime. However, as the most substantial impact is to reduce the afternoon commuting peak we assign the label to reflect this.
- *Components 5 and 6 * (Fig. 8e and f) explain only a small part of the variance (recall that components 1–3 explain 95%), and it becomes difficult to distinguish a noticeable activity that they might be associated with. We include them here as an example but the later discussion will concentrate on earlier components.

**Fig. 9:**
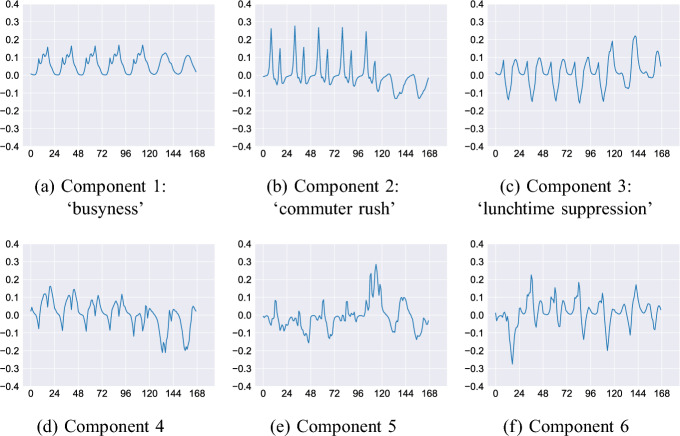
Principal components for weekly activities

The weekly aggregation gives further insight into the usage patterns with in the city, as illustrated in Fig. 9. Interestingly components 1–3 are almost identical to the first three components in the daily aggregation, but carry with them additional information particularly for the weekend.

- *Component 1, ‘busyness’ * (Fig. 9a). As with the daily aggregation, this appears to be almost identical to the mean weekly activity in Figure 5b.
- *Component 2, ‘commuter rush’ * (Fig. 9b). This component exhibits clearly identifiable early morning and late afternoon peaks during weekdays. Interestingly, during weekends there is a small but noticeable suppression of activity during the middle of the day, which gives further evidence that this component is capturing weekly commuting behaviour.
- *Component 3, ‘lunchtime suppression’ * (Fig. 9c). As with the daily aggregation, this component illustrates a suppression of footfall around lunchtime. Now the component also captures an increase in late evening activity on Friday, Saturday, and Sunday.
- *Components 4, 5 and 6 * (Fig. 9d, e, f) explain only a small part of the variance and do not discern an obvious footfall pattern.

Having calculated and described the main principal components that characterise daily and weekly footfall behaviour, the following section analyses the component loadings in more detail and discusses the insights they can provide into urban dynamics more broadly.

### Component loadings in the case study locations

**Fig. 10:**
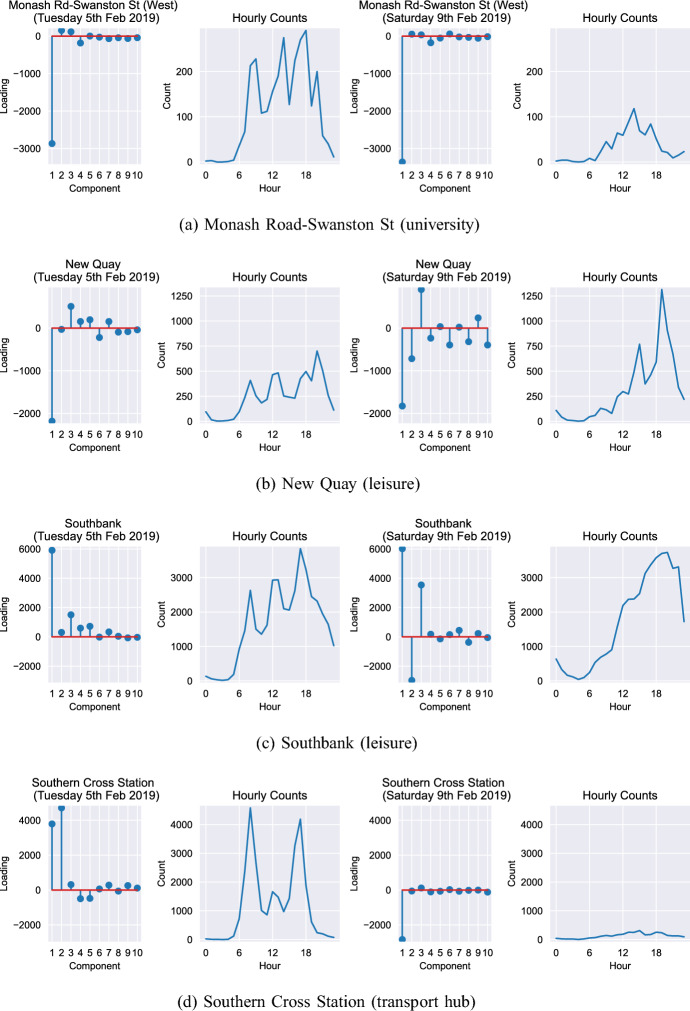
Component loadings for the four case study locations on two different days: Tuesday 5th and Saturday 9th February 2019. Only the first ten components are shown for clarity

Section 4.3 outlined four distinct locations that are useful for demonstrating the insight that can be gained through deeper analysis of the principal components. These locations are: Monash Road–Swanston Street (university); New Quay (leisure); Southbank (leisure); and Southern Cross (transport hub). In the following analysis, we illustrate how the component loadings differ for each of these locations on two specific days: Tuesday 5th and Saturday 9th February 2019. We choose a Tuesday and a Saturday to compare weekly and weekend activities and choose a week in February because this is during the summer term at Melbourne University (so student activities at the University will feature in the analysis). Figure 10 illustrates the loadings for those four locations on those 2 days.

We highlight the results for Southbank (Fig. 10c) and Southern Cross Station (Fig. 10d) as these are the most striking. Considering Southbank, on the Tuesday, the most substantial impact is caused by Component 1 (busyness). Footfall patterns at the station follow a ‘typical’ pattern with morning and evening commutes and activity at lunch time. The pattern on Saturday, however, is very different. The area remains busy on the whole, but there is a considerable decrease in component 2 activity (‘commuter rush’) to be replaced by component 3 (‘lunchtime suppression’). A secondary impact of component 3 is to increase footfall in the evenings, so ultimately it appears that on Saturday the activity in the Southbank area changes noticeably from commuting behaviour to afternoon/evening activities. With respect to Southern Cross Station, the results are even more striking. The large commuting activities that take place on the Tuesday are almost non-existent on the Saturday, evidenced by a substantial drop in the ‘busyness’ and ‘commuter rush’ component weightings.

## Results: an insight into pedestrian dynamics with PCA

### Relationships between component loadings

**Fig. 11:**
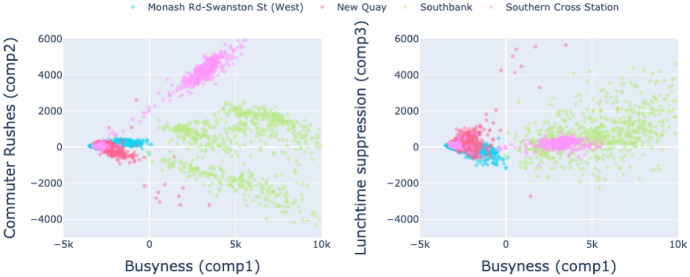
Interactions between the three main principal components in the case study locations

An advantage of deriving interpretable principal components is the ability to examine how the loadings of different components vary across observations to gain insights into urban activities. While the components themselves are orthogonal by design, their loadings can still reveal how different temporal patterns manifest across locations. To this end, Appendix A provides a three-dimensional analysis of the values of the three most important components for every sensor and every day. Interpreting all points simultaneously in three dimensions is challenging, so Fig. 11 presents the component interactions for the four case study areas in isolation, represented using two 2D plots. There are some striking features that offer insight into the nature of the four areas:

*Monash Road–Swanston Street (university)*: –*busyness* has no relationship with *commuter rushes*, but appears to have a negative relationship with *lunchtime suppression*. This implies that when the area is busier this can be attributed to an increase in footfall in the middle of the day rather than during commuting times (a negative weighting to ‘lunchtime suppression’ will *increase* footfall during the day). This observation makes sense given that the sensor is located on the Melbourne University campus.
*New Quay (leisure)*: –Increases in busyness appear to show a strong negative relationship with *commuter rushes* and no obvious relationship with *lunchtime suppression*. When New Quay is busy, this is not caused by commuters, nor are the changes restricted mainly to midday hours as was the case with Monash Road–Swanston Street.
*Southbank (leisure)*: —There is no clear relationship between *busyness* and *lunchtime suppression*, suggesting lunchtime activities do not influence busyness in a regular way (although analysis of the component loadings in Sect. 5.3 suggested an increase in *lunchtime suppression* on a Saturday, this may be obscured as it is only present on one day per week, whereas commuting behaviour is much more common). Regardless, there is a striking relationship between *busyness* and *commuter ruses* (also noticed in Sect. 5.3) that deserves further attention below.
*Southern Cross (transport hub)*: –shows an extremely strong positive relationship between *busyness* and *commuter rushes* but no relationship between *busyness* and *lunchtime suppression*, which would be expected from a public transport hub where commuting has the main influence and other components have almost *no* influence (rather than a non-systematic influence as is the case in Southbank).

**Fig. 12:**
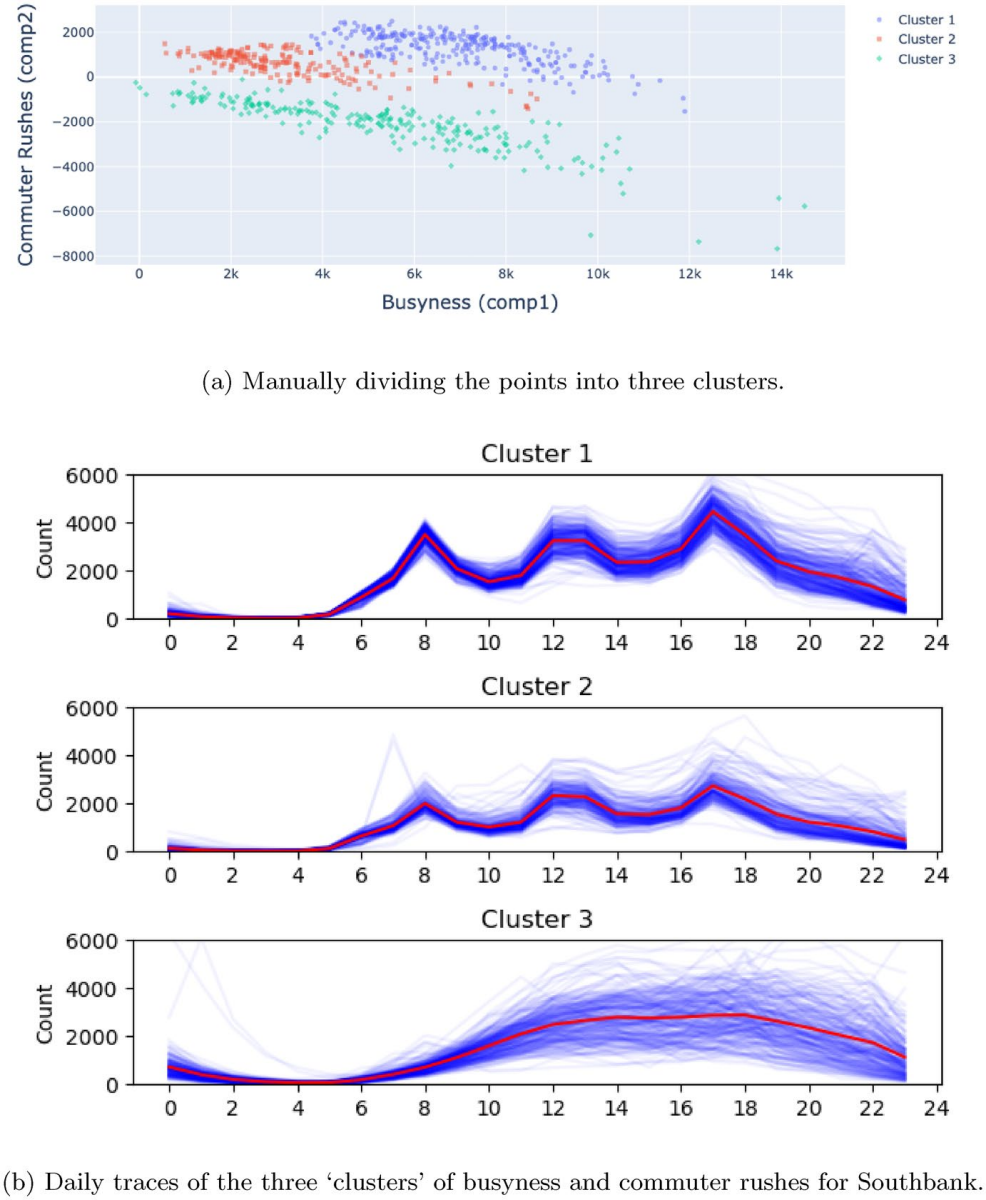
Clustering the *busyness* and *commuter rushes* components for Southbank and analysing their daily footfall traces

The relationship between *busyness* and *commuter rushes* for Southbank deserves closer attention. Firstly, however, consider again Southern Cross Station in Fig. 11. There appears to be a single, strong, positive relationship between *busyness* and *commuter rushes*. As Southern Cross Station becomes more busy, the *shape* of the daily footfall pattern does not change; we see the same footfall patterns on different days, but on some days the absolute counts are higher than on others. This is not the case with Southbank however. As Fig. 11. illustrates, there appears to be two or three separate groups of points. As with Southern Cross Station, within a single group we see the same footfall *shape*, but of varying intensity. However, the shapes within each of the three clusters will be different. In effect Fig. 11 suggests that there are two or three *different usage patterns* present in the area. To explore this further, we manually separate the groups of points into three distinct clusters and then plot the daily footfall traces for each of the clusters. Figure 12a illustrates the three manually chosen clusters and Fig. 12b illustrates their daily traces.

Observing Fig. 12, although there are small differences in the traces captured in cluster 1 and 2, broadly the daily patterns follow a typical pattern that consists of peak footfall times in the morning, lunchtime and afternoon. Cluster 3, however, captures an entirely different trend that, although exhibiting similar footfall magnitudes, exhibits a gradual increase in footfall throughout the day and does not display any discernible peaks. It is likely here that we observe substantially different weekday and weekend behaviour at Southbank, even though the footfall magnitudes are similar. In other words, the area remains busy at the weekend, but we can hypothesise that the reasons for visiting the area are different. This is in contrast to other commuting areas where the footfall magnitude diminishes at the weekend and might reveal some useful insights into the way that the built environment is used by visitors in this location.

### Exploring the evolution urban usage patterns

In this final piece of analysis, we examine longer-term footfall trends to investigate how the different patterns of behaviour vary over time. Some studies have previously investigated changes in mobility patterns due to the impact of the COVID-19 pandemic—for example: Kim and Jun (2022) analysed regional mobility shifts during COVID-19, using inflow and outflow big data to identify key patterns and influencing factors; and Schmahmann et al. (2022) examined metropolitan mobility trends, noting a decrease in inner-city movement and an increase in out-migration—but to the best of our knowledge no research has specifically used quantitative methods to explore the latent factors driving the long-term changes in footfall patterns.

**Fig. 13:**
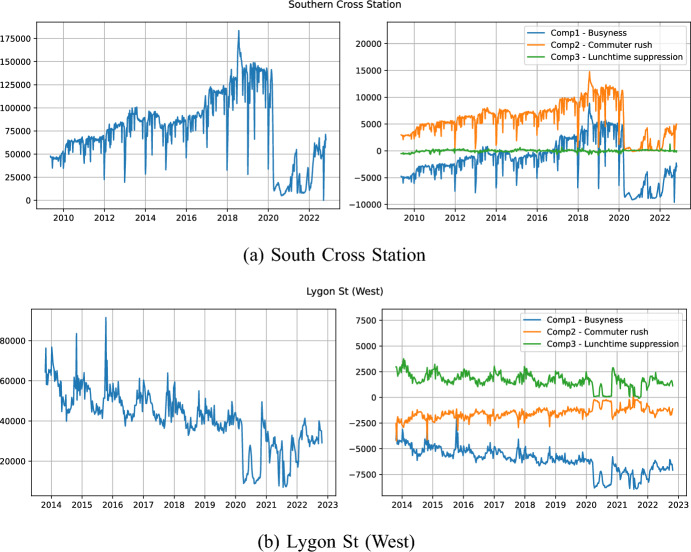
Aggregated weekly footfall counts compared with the first three components of the PCA

Having found a set of principal components using the data from 2018 and 2019, we can use these to inspect the footfall patterns at times outside this window. In Fig. 13 we present the weekly aggregated counts for two selected sites along with the first three components of the PCA decomposition. These sites are chosen specifically because they exhibit interesting patterns; in other sites there is very limited change in the longer-term trends.

For South Cross Station (Fig. 13a), we see a steady increase in pedestrian footfall between 2010 and 2020, followed by a sharp drop during the COVID-19 pandemic. Looking at the decomposition we can see that contribution due to *lunchtime suppression* remains small during the entire time, while the change only comes with the busyness and commuter rush components. Although footfall at the site appears to be increasing, it is a long way from its pre-COVID levels which may reflect larger numbers of people working from home after the pandemic and hence not returning to the area. This is potentially valuable insight for policy makers as it suggests that additional footfall might be encouraged through *secondary* means, such as making on-site working more popular to encourage greater commuting, rather than through direct initiatives that improve the attractiveness of the area.

Considering Lygon Street (Fig. 13b), we see a steady decline in footfall between 2014 and 2022, which becomes more chaotic during COVID-19. Considering the PCA components, we can see that this decline has been driven by a decrease in overall *busyness* and a small decline in lunchtime suppression, while *commuter rushes* have actually increased over time. This suggests that although Lygon St. is gradually becoming less busy, it may be transitioning into a more commuter-oriented location.

## Conclusions, limitations and future work

This paper has demonstrated how the use of principal component analysis (PCA) can lead to insight into the key temporal signatures that underpin urban footfall. By using PCA, it is possible to reduce a complex, noisy time-series to a more comprehensible number of components, and then analyse these components to draw inferences about urban space usage more broadly. This final section will discuss the limitations of the work and outline avenues for future research.

An obvious drawback with the approach relates to the validation of the identified activities. Although the components that we extract *appear* to be related to well-known activities, such as ‘9–5’ commuting, we do not have any information about the activities that the individuals who contribute to the footfall counts are actually doing. Although the patterns match expectations and findings in previous work, such as the ‘three peaks’ identified by others (Kim 2020; Dobler et al. 2021), and it is difficult to imagine an alternative explanation for these structures, further work would be needed to validate these assumptions. There are several possible avenues for validation. For example, it could involve the use of activity surveys in the region, either via a new, bespoke survey, or through the analysis of existing mobility surveys such as the UK National Travel Survey that collects information on “how, why, when and where people travel”(Department for Transport 2024). Alternatively, validation could be achieved through triangulation with alternative data-driven mobility estimates, such as the LandScan or Population 24/7 products (see Richardson (2020) for further detail) or via a more nuanced analysis of individual movement traces, such as those that are created passively through mobile phone use.

A second issue relates to bias and representation. Although Melbourne has a very large number of publicly available footfall sensors—the largest number for a city available globally as far as we are aware—we have not attempted to analyse the equity in their spatial distribution. Evidence from other places suggests that sensors such as these are not typically distributed across the population equitably (Robinson and Franklin 2020; Robinson et al. 2022) and instead cluster in areas that are well known by the researchers tasked with deploying them or in places where, for a variety of reasons, their deployment is relatively straightforward from an administrative/infrastructure perspective. The result is that, unless the sensor locations have been carefully planned, they are unlikely to provide a ‘true’ picture of the variety in footfall patterns that occur throughout the city. Future work could attempt to explore this question by analysing the demographics of the city more broadly and assessing the degree to which the sensor locations capture the behaviour of *all* residents, although this will of course be complicated by the fact that while the sensors measure *outdoor* activity, most demographic data sets will capture *residential* characteristics. Again, qualitative surveys of the people who visit different parts of the city might help.

The generalisability of the work comes in to question because we do not attempt to apply the method to places other than Melbourne, Australia. That said, the patterns that we uncover are not especially surprising; they are similar to those found cities such as New York (Dobler et al. 2021) and Seoul (Kim 2020) so may well be representative of other major cities in ‘Global North’ countries. Data permitting, it would be interesting to apply the method in a variety of different places to determine whether the same key footfall components can explain such a large part of the overall variance. Similarly, we base most of the analysis on data for the 2018–2019 period in order to avoid the huge disruption to footfall patterns that occurred during the COVID-19 pandemic. Although unlikely, as there is nothing to suggest that 2018–2019 was an exceptional period in Melbourne’s history (unlike, say, 2020), it is possible that had the key temporal signatures might be different were another time period chosen. A related issue is that while PCA effectively uncovers regular temporal patterns in footfall data, it lacks the capability to detect anomalies and special events. Here we have focussed on more regular ‘signatures’, but future work will aim to complement the current analysis with methods that enhance the identification of such variations.

In conclusion, in this paper we apply the technique of principal component analysis (PCA) to footfall count data from Melbourne, Australia. Interestingly the three components that explain most of the variability in the footfall patterns across the city are recognisable as typical activity patterns, such as ‘9–5’ commuting or leisure activities during evenings and weekends. Further analysing the components themselves, and their interactions, offers the opportunity to better understand the dynamics of pedestrian behaviour in the city from a data-driven/quantitative perspective to complement qualitative work. Immediate plans for future research include (i) assessing the generalisability of the work by replicating the study across a larger number of cities and (ii) exploring the demographics of the city in more detail to better understand the cause of the observed patterns.

## Figures and Tables

**Fig. 14:**
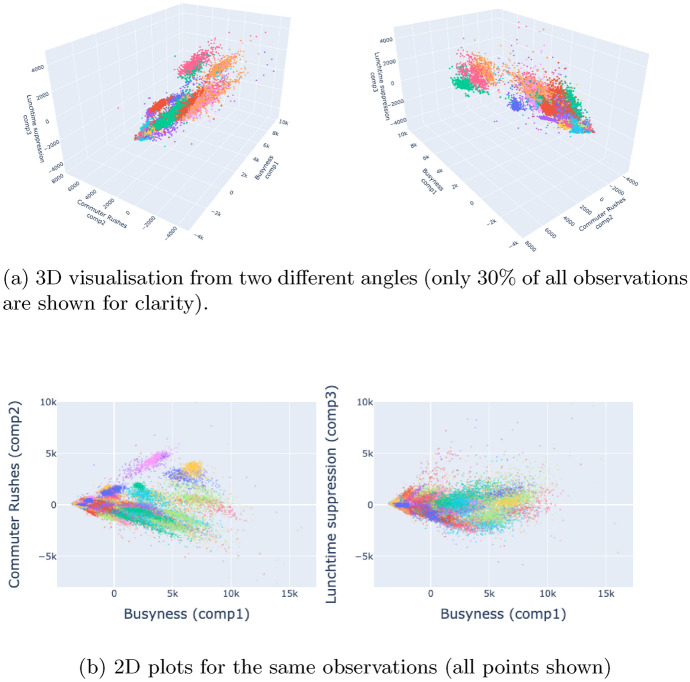
Visualisation of the first three principal components for every sensor and day. Colours are chosen randomly and uniquely to represent the different sensors (colour figure online)

## Data Availability

The code and data that underpin the work in this paper are all publicly available as follows: The underlying pedestrian count data are available from the City of Melbourne Open Data Portal, specifically the ‘Pedestrian Counting System’: https://data.melbourne.vic.gov.au/explore/dataset/pedestrian-counting-system-monthly-counts-per-hour/information/. The full source code is publicly available at the following GitHub repository: https://github.com/nickmalleson/melbourne-timeseries. The code repository at the point of paper acceptance has been tagged and assigned the following DOI: https://doi.org/10.5281/zenodo.15297542. The code has been designed to download the required data on first use. However, in case the data cannot be obtained in the future, a full copy of the code repository *including the required data* has been archived with Figshare at: https://dx.doi.org/10.6084/m9.figshare.28882904.

## Appendix A: Interpretation of the components in 3D

Figure 14 illustrates the values of the three most important components (busyness, commuter rushes and lunchtime suppression) for ever sensor and every day. Note that interpretation of the 3D plot is considerably easier when these are viewed interactively so that they can be zoomed and rotated. To this end, an html file has been included in the Supplementary Information that provides a 3D interactive plot that allows the reader to explore the point cloud in more detail.

The most striking immediate observation from Fig. 14 is the *cone* shape: as the busyness component increases there is a much greater spread in the values of the other two components (both positive and negative). This is interesting but not unexpected; if the value of the first component is large, then the other components must necessarily have particularly large or small values to have a noticeable impact on the final footfall counts. More broadly, the analysis of individual sensors using interactive 3D plots such as this could produce unexpected insights into the dynamics of urban footfall.

## Footnotes

1 The Melbourne footfall data are available publicly through the Melbourne Open Data Portal: https://data.melbourne.vic.gov.au/explore/dataset/pedestrian-counting-system-monthly-counts-per-hour/information/

2 A Doppler radar system is likely two reasons. Firstly, the sensor location contains two *direction* fields and there is also a data set aggregated by minutes which contains the counts in two different directions.
